# Green Extraction Technology of Polyphenols from Food By-Products

**DOI:** 10.3390/foods11081109

**Published:** 2022-04-13

**Authors:** Anastasia Kyriakoudi, Ioannis Mourtzinos

**Affiliations:** Laboratory of Food Chemistry and Biochemistry, Department of Food Science and Technology, School of Agriculture, Faculty of Agriculture, Forestry and Natural Environment, Aristotle University of Thessaloniki (AUTH), 54124 Thessaloniki, Greece; ankyria@agro.auth.gr

The development of environmentally friendly approaches to produce high-added value compounds is a field of research that has attracted the interest of the scientific community and several industries such as the food and cosmetic industry. The last decades, there has been an increasing demand for foods that not only have high nutritional and sensorial quality, but also deliver health-promoting benefits through certain ingredients, namely “bioactive” or “functional” ones. Moreover, emphasis is given on the production of healthy products without the use of synthetic chemical food additives. In this way, and in order to protect both public health and the environment, food by-products, generated in large amounts worldwide, can be exploited as a promising source of valuable compounds, such as polyphenols [1]. In this direction, this Special Issue (SI) entitled “Green Extraction Technology of Polyphenols from Food By-Products” focuses on green approaches for the recovery of polyphenols from different food by-products. The present SI consists of six research articles and one review article that contribute to the objectives of *Foods*.

In particular, Fia et al. (2020) [2] examined the recovery of bioactive compounds from unripe red grapes (cv. Sangiovese) which constitute a by-product of the wine industry. The authors proposed an industrial-scale green extraction method employing maceration without the use of solvents for the preparation of an extract with a high concentration of bioactive compounds. The prepared extracts could find applications as functional ingredients or as natural additives (e.g., antioxidants) in foods and beverages.

Kyriakidou et al. (2021) [3] investigated the incorporation of pomegranate peel extracts, obtained by deep eutectic solvents, in chitosan films. Pomegranate peels are a rich source of phenolic compounds, such as punicalagin, gallic acid, and ellagic acid. In this study, the authors investigated the physicochemical properties of chitosan-based edible films containing pomegranate peel extracts as plasticizers.

The potential for the exploitation of apple pomace, the main by-product derived during apple processing, as a source of bioactive compounds, that could be used in the food industry, was studied by Liu et al. (2021) [4]. The authors investigated the biotransformation of polyphenols in apple pomace fermented by β-glucosidase-producing *Lactobacillus* strains in order to liberate the aglycone from bound forms of phenolics.

The extraction of thinned unripe peach (*Prunus persica* L. cv. Hujingmilu) polyphenols using ultrasounds as well as their purification through a macroporous resin, was examined by Dai et al. (2022) [5]. The authors concluded that the purified thinned peach polyphenols exhibited antioxidant, hypoglycemic, and hypolipidemic capacity, and could be further exploited apart from their use as poultry feed. 

The ultrasound-assisted extraction of polyphenols from the pulp of ripe carob pods (*Ceratonia siliqua* L.) was examined by Clodoveo et al. (2022) [6]. The extraction process was optimized in terms of solid:solvent ratio, solvent concentration, and particle size. The authors suggested that carob pod pulp is a natural source of polyphenols and proposed the valorization of this agri-food waste.

Kirindage et al. (2022) [7] examined the efficacy of hot water to extract bioactive compounds from *Moringa oleifera* and evaluate their antioxidant activity. The authors concluded that the prepared extracts were found to reduce the intracellular generation of reactive oxygen species in H_2_O_2_-stimulated Vero cells in a dose-dependent manner, and that they could find applications in the development of functional foods.

Clodoveo et al. (2022) [8] critically reviewed recent advances in novel extraction technologies for the development of functional ingredients based on polyphenols from olive leaves. The latter ones constitute one of the main by-products derived from olive tree cultivation and the olive processing industry that is usually discarded, causing economic and environmental problems. The authors focused on the major phenolic compounds that are present in olive leaves, the innovative extraction technologies, as well as potential industrial uses, and international patents that have been filed in the food, pharmaceutic, and cosmetic sectors.

## References

[1] Mourtzinos I., Goula A., Watson R.R. Polyphenols in agricultural by-products and food waste. Polyphenols in Plants-Isolation, Purification and Extract Preparation.

[2] Fia G., Bucalossi G., Gori C., Borghini F., Zanoni B. (2020). Recovery of Bioactive Compounds from Unripe Red Grapes (cv. Sangiovese) through a Green Extraction. Foods.

[3] Kyriakidou A., Makris D.P., Lazaridou A., Biliaderis C.G., Mourtzinos I. (2021). Physical Properties of Chitosan Films Containing Pomegranate Peel Extracts Obtained by Deep Eutectic Solvents. Foods.

[4] Liu L., Zhang C., Zhang H., Qu G., Li C., Liu L. (2021). Biotransformation of Polyphenols in Apple Pomace Fermented by β-Glucosidase-Producing *Lactobacillus rhamnosus* L08. Foods.

[5] Dai K., Wei Y., Jiang S., Xu F., Wang H., Zhang X., Shao X. (2022). Lab Scale Extracted Conditions of Polyphenols from Thinned Peach Fruit Have Antioxidant, Hypoglycemic, and Hypolipidemic Properties. Foods.

[6] Clodoveo M.L., Crupi P., Muraglia M., Corbo F. (2022). Ultrasound Assisted Extraction of Polyphenols from Ripe Carob Pods (*Ceratonia siliqua* L.): Combined Designs for Screening and Optimizing the Processing Parameters. Foods.

[7] Kirindage K.G.I.S., Fernando I.P.S., Jayasinghe A.M.K., Han E.-J., Dias M.K.H.M., Kang K.-P., Moon S.-I., Shin T.-S., Ma A., Ahn G. (2022). *Moringa oleifera* Hot Water Extract Protects Vero Cells from Hydrogen Peroxide-Induced Oxidative Stress by Regulating Mitochondria-Mediated Apoptotic Pathway and Nrf2/HO-1 Signaling. Foods.

[8] Clodoveo M.L., Crupi P., Annunziato A., Corbo F. (2022). Innovative Extraction Technologies for Development of Functional Ingredients Based on Polyphenols from Olive Leaves. Foods.

